# Spin-orbit interaction driven terahertz nonlinear dynamics in transition metals

**DOI:** 10.1038/s44306-024-00068-7

**Published:** 2025-01-27

**Authors:** Ruslan Salikhov, Markus Lysne, Philipp Werner, Igor Ilyakov, Michael Schüler, Thales V. A. G. de Oliveira, Alexey Ponomaryov, Atiqa Arshad, Gulloo Lal Prajapati, Jan-Christoph Deinert, Pavlo Makushko, Denys Makarov, Thomas Cowan, Jürgen Fassbender, Jürgen Lindner, Aleksandra Lindner, Carmine Ortix, Sergey Kovalev

**Affiliations:** 1 Helmholtz-Zentrum Dresden-Rossendorf, Dresden, Germany; 2 Department of Physics, University of Fribourg, Fribourg, Switzerland; 3 Laboratory for Materials Simulations, Paul Scherrer Institute, Villigen, Switzerland; 4 Technische Universität Dresden, Dresden, Germany; 5 Dipartimento di Fisica “E.R. Caianiello”, Universita di Salerno, Fisciano, Italy; 6 Fakultät Physik, Technische Universität Dortmund, Dortmund, Germany

**Keywords:** Spintronics, Terahertz optics

## Abstract

The interplay of electronic charge, spin, and orbital currents, coherently driven by picosecond long oscillations of light fields in spin-orbit coupled systems, is the foundation of emerging terahertz lightwave spintronics and orbitronics. The essential rules for how terahertz fields interact with these systems in a nonlinear way are still not understood. In this work, we demonstrate a universally applicable electronic nonlinearity originating from spin-orbit interactions in conducting materials, wherein the interplay of light-induced spin and orbital textures manifests. We utilized terahertz harmonic generation spectroscopy to investigate the nonlinear dynamics over picosecond timescales in various transition metal films. We found that the terahertz harmonic generation efficiency scales with the spin Hall conductivity in the studied films, while the phase takes two possible values (shifted by π), depending on the *d*-shell filling. These findings elucidate the fundamental mechanisms governing non-equilibrium spin and orbital polarization dynamics at terahertz frequencies, which is relevant for potential applications of terahertz spin- and orbital-based devices.

## Introduction

The interaction between terahertz (THz) light and matter has gained significant attention due to its potential for the coherent generation and nonlinear manipulation of charge and spin currents on ultrafast timescales. Notable examples include THz-driven Dirac currents in topological surfaces^1^, efficient THz frequency conversion^2,3^, ultrafast magnetization switching^4^, and short-wavelength magnon excitation via spin-orbit torques^5^. Extensive research is being conducted in the areas of nonlinear THz-frequency dynamics in superconductors^6,7^, Dirac materials^8,9^, and magnetic heterostructures^10–12^. The THz field-driven nonlinear processes can arise from various phenomena, such as electronic phase transitions that lead to enormous conductivity changes in superconductors^6,7^, the non-parabolic band structure of carriers in Dirac materials^3,8,9^, and the low heat capacity in graphene necessary for thermodynamic harmonic generation^2^. There is intense research focused on utilizing ultrafast spin-to-charge interconversion, which exploits spin-pumping effects at magnetic/nonmagnetic interfaces as a means of THz harmonic generation^10,11^. Despite significant progress in the research on nonlinear THz-driven dynamics in complex systems, a comprehensive understanding of the interaction between THz light and metals has yet to be established.

It is believed that metallic films exhibit negligible THz nonlinearities due to parabolic electronic band structure, and large electronic heat capacity. The free electron dynamics in metals shows a linear^13^ or thermal^14^ response at THz frequencies. This holds true as long as the electronic spin and orbital degrees of freedom are not considered. The spin-based non-equilibrium processes in transition metals (TMs) are central to the operation of spintronics devices^15,16^. The charge-to-spin conversion via the spin Hall effect and the reciprocal spin-to-charge conversion via the inverse spin Hall effect in TMs remain efficient even at picosecond time scales^5,17–20^. Additionally, recent studies have reported on ultrafast orbital angular momentum currents, which are then converted into charge currents via the inverse orbital Hall or orbital Rashba-Edelstein effects^21–25^. These spin-based phenomena have recently been leveraged for THz-driven nonlinear processes at ferromagnet/heavy metal (FM/HM) interfaces, where the interface acts as a rectifier of THz-field induced electric currents^26^. Additionally, ultrafast unidirectional spin Hall magnetoresistance has been experimentally observed. In this effect, spin accumulation at the FM/HM interface, induced by the spin Hall effect, leads to resistivity modulation depending on the spin orientation relative to the direction of magnetization in the FM layer, resulting in a magnetoresistance-like effect^27^. Both phenomena contribute to THz second harmonic generation and THz rectification processes, establishing nonlinear THz spectroscopy as an insightful method for studying spin Hall effect-related phenomena on ultrafast time scales.

In this work, we reveal a nonlinear THz response in TM films, which originates from ultrafast electron spin dynamics coupled to the non-equilibrium orbital accumulation. The coupling results in terahertz third harmonic generation (THG), as demonstrated in various 3 *d*, 4 *d*, and 5 *d* TMs. We show that the THG amplitude closely resembles the spin Hall conductivity (SHC) values of the studied films, and the THG phase displays two possible values depending on the sign of the SHC. Our research provides new insights into the general and complex interplay between the nonlinear dynamics of charge carriers, their spin and orbital momenta, which is important for understanding non-equilibrium manipulation of matter.

## Results

**Fig. 1:**
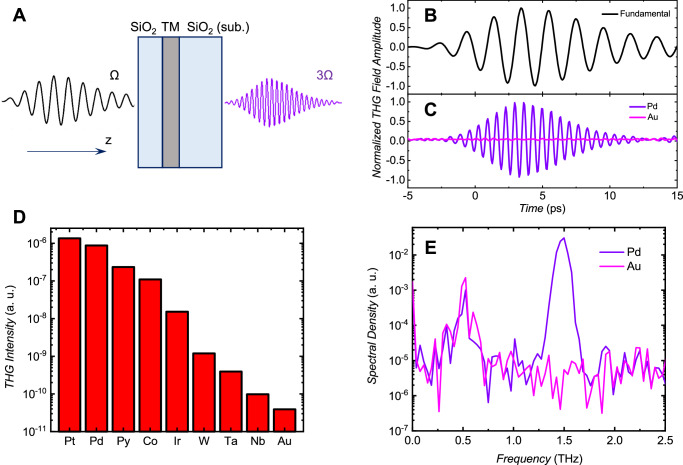
Third harmonic generation in transition metals. **A** Schematics of the experiment. Multi-cycle terahertz pulses with a central frequency of Ω (black) are incident on TM films capped with SiO_2_. The emission of terahertz radiation at the third harmonic frequency, 3 Ω (violet), is detected using electro-optical sampling. **B** Time scan of the incident (fundamental) radiation, measured directly from the source. **C** Comparison of the THG signals between 4 nm-thin Pd and Au layers. The THG signal from the Pd layer is evident, while the Au layer in our samples exhibits virtually zero THG amplitude. The fast Fourier transform spectra of the corresponding raw signals (provided in Fig. S1), are shown in **(E)**. The leaking signal from the fundamental beam is visible at 0.5 THz for both samples. Only the Pd layer shows an intense signal at the third harmonic of 1.5 THz. **D** The comparison of the THG signal intensity, normalized to the cube of the sample’s transmission (at 0.5 THz), for various transition-metal films. To ensure consistency, the transmitted fundamental and THG signals from all films were measured under the same experimental conditions. Please, note the logarithmic scale.

To investigate the nonlinear carrier dynamics in transition metal films, we utilized THz time-domain spectroscopy (TDS). The schematic of our experiment is illustrated in Fig. 1A. We employed a narrowband THz radiation (referred to as fundamental beam) with a central frequency of 0.5 THz (unless otherwise specified), a bandwidth of about 20%, and a pulse energy of 1.5 µJ (details of the fundamental beam source are provided in the Methods). Figure 1B shows the time profile of the fundamental radiation with an estimated peak field amplitude of about 200 kV/cm. The radiation was focused on a sample, which consisted of a 4 nm-thin (unless otherwise specified) TM film (nonmagnetic - Pt, Pd, Ir, W, Ta, Nb, Au and magnetic - Co, Py = Ni_81_Fe_19_), grown on a 1 mm thick quartz glass substrate using magnetron sputter deposition. All samples were capped by a 10 nm-thick SiO_2_ layer, unless otherwise specified. The generated (and transmitted through the sample) THz radiation at 1.5 THz was refocused on a 2-mm thick ZnTe crystal, and its dynamics was measured using electro-optic sampling. Two bandpass filters with a central frequency of 1.5 THz were placed between the sample and ZnTe crystal to suppress the fundamental radiation. However, leakage of the fundamental radiation was observed in all experiments. Therefore, for further analysis, we employed a digital high-pass filter with a 1 THz cut-off frequency.

Figure 1C presents the processed THz THG data for Pd and Au samples. The time delay scans affected by fundamental beam leakage are shown in Supplementary Materials Fig. S1. The Pd film serves as a source for THz THG, whereas no harmonics are observed in the Au sample. This is further confirmed by examining the raw data Fourier spectra shown in Fig. 1E. The Pd spectra exhibits the fundamental beam leak signal at 0.5 THz and the THG at 1.5 THz. Conversely, only the leak signal is detected from the Au film. The efficiency of THG in Pd, measured at room temperature, is about 10^-4^ in field amplitude, which is approximately two orders of magnitude smaller than that observed in topological insulators^3^. The THG signal is linearly polarized, aligned with the polarization of the fundamental beam, and disappears for circular polarization (see Supplementary Materials, Fig. S2).

Figure 1D presents the THz THG intensity collected for all samples. To ensure a fair comparison, the THG intensity was normalized by the cube of each sample’s transmission measured at 0.5 THz. The THG intensity is highest for the platinum sample, lowest for the niobium sample, while the gold sample shows no THG signal. The THG signal in the gold film is determined by the measurement noise floor and is similar to that of the bare substrate, representing the sensitivity limit of our experimental setup. We found no impact of magnetic fields with amplitude up to 1 T and direction within the film plane on the THG signal in all samples. The THG of magnetic films such as Py and Co was found to be independent on their magnetization direction, as demonstrated in Supplementary Materials, Fig. S3.

The distribution of the THG intensity across different materials exhibits a qualitative correlation with their SHC characteristics^28^. Similar to the SHC, the THG intensity (Fig. 1D) is found to be the highest for Pt, Pd and Py (Py = Ni_81_Fe_19_), which belong to the same group-10 elements of the Periodic Table. When moving from the 3 *d* (Py) to the 4 *d* (Pd) and 5 *d* (Pt) elements, the THG intensity increases. This trend is in line with the SHC, which is consistent with the known increase in spin-orbit coupling strength with atomic number^15,28–30^. As one moves through the series of 5 *d* elements (Ta, W, Ir, Pt, Au), the THG intensity distribution in Fig. 1D closely resembles the SHC calculations^28–30^. When considering ferromagnetic metals (Py and Co), it is predicted that Py has a larger SHC compared to Co^28^, and this trend is also evident in our diagram in Fig. 1D. Please note that conducting a quantitative comparison with experimental data is challenging due to the wide variability in SHC across experimental studies. This variation can result from differences in measurement methods, growth conditions, sample quality, and interfacing with other films^15,30^. However, we proceed to demonstrate that the THG phase exhibits two possible values depending on the sign of the SHC. This further reinforces our conclusion regarding the spintronic origin of the nonlinear effect.

**Fig. 2:**
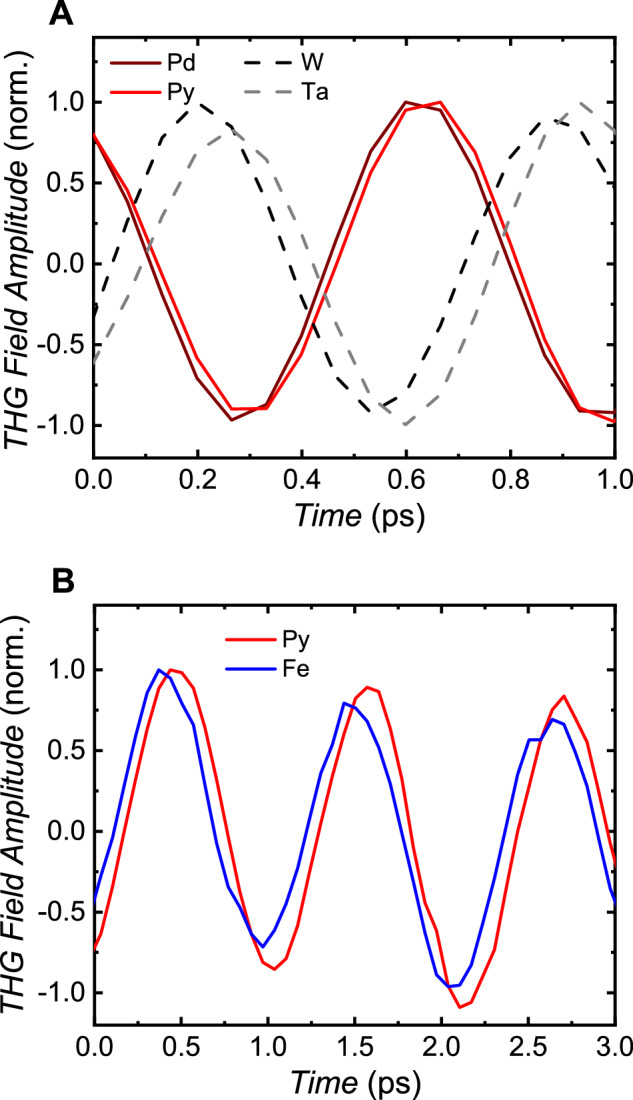
Third harmonic radiation phase. **A** Comparison of THG time scans for different transition-metal films; **B** 4 nm-thin Fe (bcc) and Py (fcc) films grown on MgO substrates using molecular beam epitaxy and capped with a 3 nm thin Au layer. The films in **(B)** were excited using a 0.3 THz fundamental beam. TMs with negative SHC or less than half-filled *d*-bands are indicated with a dashed line.

Next, we illustrate that the THG phase depends on the *d*-band filling and, consequently, on the sign of the SHC. To demonstrate this relationship, two sets of experiments were conducted using different samples. The results are shown in Fig. 2. Figure 2A compares time scans of the THG signal in the samples. There is a phase shift of about 180 degrees between the Pd and Py group of samples (solid lines) and the Ta and W group (dashed lines). The THG signals of the Pt, Ir, Co, Py (Nb, Ta) films are in phase with that of Pd (W). Note that Nb, Ta, and W have less than half-filled *d*-shells and exhibit a negative SHC^28–30^. To eliminate the effect of the crystal structure, we compared the THz THG phase in the body-centered cubic (bcc) Fe film and in the face-centered cubic (fcc) Py film, both grown using molecular beam epitaxy (see Methods for details). Both samples were covered with an Au layer to protect them from oxidation. We observed no significant changes in the THG phase within the limits of experimental accuracy (Fig. 2B), suggesting that the THG phase does not categorize the studied TM films based on their crystal structure. We further note that within a range of TM thicknesses from 2 nm to 12 nm, neither the transmitted fundamental signal (Supplementary Materials, Fig. S4) nor the THG signal (Supplementary Materials, Fig. S5) demonstrate any visible change in their phase. Additionally, the THG phase for all samples remains unchanged when using THz fundamental radiation with a central frequency of 0.3 THz. (Supplementary Materials, Fig. S6).

**Fig. 3:**
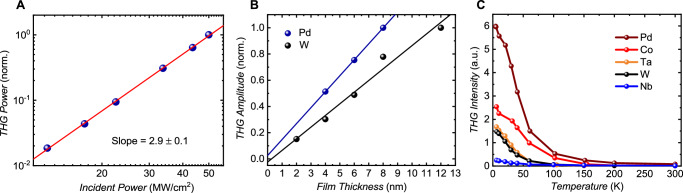
Impact of incident power, layer thickness and temperature. **A** Incident power dependence of the third harmonic signal in a Pd film. The red solid line shows the linear fit to the data in the log-log plot. **(B)**, The effect of Pd and W film thickness on the third harmonic generation amplitude (normalized), after the correction of the screening effect. Solid lines represent linear fits to the data points. **(C)**, Temperature dependence of terahertz harmonic generation in various 4 nm-thick transition metal films. The data in (C) do not account for the screening effect; no normalization based on the sample’s transmission was performed.

We also studied THz THG dependence on the fundamental beam fluence, film thickness, and temperature. As shown in Fig. 3A, the THG intensity follows the expected proportional relationship to the cube of the fundamental power, with no saturation even at incident field of 200 kV/cm. The cubic dependence reflects the order of the nonlinear process, and the absence of deviation from this dependence indicates that access to the charge, spin, and orbital carriers is not limited at THz fields up to 200 kV/cm, as expected for metallic samples. To examine the thickness dependence, we used Pd and W samples of varying thicknesses, each capped with a SiO_2_ layer (Fig. 3B). We normalized the THG intensity to the cube of the sample transmission to account for screening effects (the data without normalization are given in the Supplementary Materials, Fig. S7). The THG field amplitudes in both samples exhibit a linear behavior with thickness (thicknesses in the order of 10’s nm are considered). This behavior suggests that the THG processes does not come about pure surface contributions^12,31,32^. At the Pd film thickness of 2 and 12 nm, no visible THG was detected. The THz THG originating from the surface states of elemental Bi exhibits a phase shift of 90 degrees compared to the THz THG in transition metals (see Supplementary Materials, Fig. S8). This further suggests a THG mechanism different from that due to Rashba surface states.

The temperature dependence of THz THG in Pd, Co, Nb, Ta and W films was studied using a gas flow cryostat. Due to the limitations of the cryostat’s numerical aperture, the field strength of the pump pulse was reduced, resulting in only the Pd and Co films showing THG above the noise floor at room temperature. Nevertheless, all TM films demonstrated a strong increase in THz THG as temperature decreased (Fig. 3C). The THG in the Pd sample was observed to be over 60 times higher at 5 K than at 300 K. The phase of THG was found to be temperature-independent. Note that in Fig. 3C the THz THG intensity is plotted without normalization based on the samples’ transmission.

## Discussion

The intensity and phase of harmonic generation in various TMs resemble their spin Hall characteristics, suggesting a spintronic origin for the nonlinear interactions between THz light and metallic films. Before delving into an explanation for this effect, we first summarize our experimental observations.

1. THG is observed across various transition metal films, spanning 3 *d*, 4 *d*, and 5 *d* elements. The THG phase exhibits two possible values, shifted by π depending on the *d*-shell filling. Notably, the THz THG amplitude and phase remain unaffected by applied magnetic fields (up to 1 T), magnetization direction (for Co, Fe, and Py), and crystal structure (bcc Fe versus fcc Py). Furthermore, the THG phase remains independent of temperature and thickness.
2. The amplitude of THG in various samples correlates with their SHCs, with the highest observed for Pt and the smallest for Nb. Further details on the correlation between THG amplitude and SHC are provided above. The observation that the THG phase reflects the *d*-shell filling further bolsters the spintronic nature of the effect, as the sign of the SHC also depends on the *d*-shell filling. Interestingly, our polycrystalline Au film does not exhibit any visible THG within the signal-to-noise level. This aligns with the negligible intrinsic spin Hall contribution in Au films^33^. We note that the significant SHC in Au is primarily attributed to surface scattering^34^. This suggests that surface effects are not expected to yield any noticeable contribution in our experiment.
3. Indeed, the linear thickness dependence shown in Fig. 3B for Pd and W suggests that the THG observed in our samples does not originate from spin-orbit coupled surface states. Additionally, we show in the Supplementary Information that THG from surface states is not expected to generally display a π phase shift. The filling of the *d* bands is reflected in a change of the Rashba coupling characterizing the surface states. This does neither change the carrier velocities nor the geometrical properties of the electronic wave-functions affecting nonlinear electromagnetic properties, including the Berry connection polarizability entering the third-order current response.
4. The intensity of THG significantly increases as the temperature decreases from 300 K to 5 K, resulting in a 60-fold increase in Pd (see Fig. 3C).

In terms of symmetry, the majority of metallic films under study typically exhibit a cubic structure, resulting in electron states (Bloch functions) that display symmetry upon inversion of coordinate space (even parity of *s*- and *d*-orbital atomic wave functions in fcc and bcc TMs). When combined with a translation of time by half the period of the THz field (represented as X(t) = X(t + T/2), where X denotes the electron response function, t is time, and T is the THz period), this phenomenon leads to the suppression of even harmonics and the presence of odd harmonics^35^. Our numerical simulations based on relativistic density functional theory calculations (see Supplementary Materials), have successfully illustrated the phenomenon of THG in various TMs. However, these simulations encounter challenges in accurately reproducing the amplitudes of THG, and notably, they do not yield any observable phase shift when applied to Pd and W. Despite accounting for factors such as spin texture and spin-orbital coupling (which is inherent in our formalism), along with various electron relaxation times, the numerical results do not exhibit the observed π dependence on the *d*-shell filling.

**Fig. 4:**
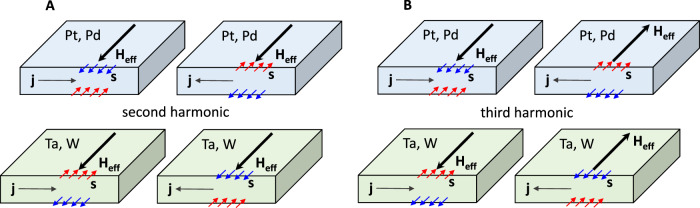
Schematic illustration of spintronic second harmonic generation and third harmonic generation. **A** Represents SHE-induced spin polarization, **s,** which interacts with a static effective field, **H**_**eff**_, resulting in a modulation of electrical conductivity, for example, due to spin-Hall magnetoresistance, and second harmonic generation. **B** Similar to (**A**), but this time the direction of **H**_**eff**_ is modulated by the direction of electrical current, j. In this scenario, the interaction between s and H_eff_ leads to third harmonic generation.

Since the observed THG in TMs cannot be straightforwardly explained by numerical calculations based on Wannier functions or by the contribution of Rashba-like surface states (as discussed in the Supplementary Materials), we propose a possible explanation using a phenomenological approach. This approach incorporates a spin-dependent symmetry to account for the THG phase shift between Pd and W TM groups. Generally speaking, nonlinear responses originate from the current-induced changes in electrical conductivity^27,36^. A good intuitive example is the second harmonic longitudinal signal resulting from unidirectional spin Hall magnetoresistance in ferromagnetic/heavy metal (FM/HM) bilayers^36^. In Fig. 4A, we illustrate the current-dependent modulation of conductivity, which arises from the magnetoresistance contribution. Here, conductivity varies based on the current direction: it increases when the spin accumulation vector ($\overrightarrow{s}$) aligns parallel to an effective field direction (here, we substitute the magnetization of the adjacent FM layer in ref. ^36^. with the ${\vec{H}}_{{eff}}$) and decreases when the spin accumulation vector aligns antiparallel to ${\vec{H}}_{{eff}}$. This modulation in conductivity ($\sigma$) can be described as $\sigma \sim \vec{s}(\Omega )\,\cdot {\vec{H}}_{{eff}}$, where ${\vec{H}}_{{eff}}$ is time independent vector^36^. Considering that the spin accumulation at the FM/HM interface in HM layers such as Pt and W has an opposite direction (due to the opposite sign in the spin Hall angle), the second harmonic signal in these heterostructures would exhibit a phase shift of π^26,36,37^. We emphasize that, in all our samples, we have not observed a second harmonic signal either in an applied magnetic field up to 1 T or in ferromagnetic films (Co, Fe, Py) with varying magnetization directions. As discussed earlier, the symmetry of THG necessitates the translation of time by half the period of the THz field^35^, indicating that the direction of ${\vec{H}}_{{eff}}$ is expected to be time-dependent, leading to: $\sigma \sim \vec{s}\left(\Omega \right)\,\cdot {\vec{H}}_{{eff}}(\Omega )$. This mechanism is schematically illustrated in Fig. 4B. It is immediately evident that due to the opposite sign of the spin Hall angle in Pd and W, the sign (and thus the THG phase) of the scalar product $\vec{s}\left(\Omega \right)\,\cdot {\vec{H}}_{{eff}}(\Omega )$ differs for the Pt and W groups of elements.

Now, the justification for ${\vec{H}}_{{eff}}$ in our model needs to be provided, considering its intrinsic and non-local character, and its direction needs to be identical for both Pd and W groups of elements as depicted in Fig. 4B. A possible candidate could at first glance be the THz field induced spin Nernst effect^28,38,39^. However, the spin Nernst conductivity (SNC) in Pt and W have opposite signs compared to their SHC^28,38,39^, while in Pd and Ta the SNC has the same sign as the SHC^28^. Hence, the spin Nernst effect cannot categorize THG phase in TMs according to their *d*-band filling. Taking into account all the requirements for ${\vec{H}}_{{eff}}$, the most plausible candidate is the intrinsic, nonlocal orbital angular momentum (orbital current) that arises from the orbital Hall effect (OHE)^21–25,28–30,40–44^. The OHE consistently manifests a positive sign across transition-metal-based materials^28–30^, which is a crucial factor in elucidating the THG phase. Similar to the spin current ${J}_{S}={\sigma }_{{SHE}}E$ (${\sigma }_{{SHE}}$, $E$ is the SHC and THz field, respectively), the orbital current ${J}_{O}={\sigma }_{{OHE}}E$ is transverse to the electrical current and satisfies the symmetry requirements for the ${\vec{H}}_{{eff}}$. Again, the orbital Hall conductivity (OHC) ${\sigma }_{{OHE}}$ is always positive, i.e. independent of the *d*-electron band filling. We thus consider the spin interaction Hamiltonian to be $(\overrightarrow{s},\overrightarrow{L}) \sim {\sigma }_{{SHE}}{\sigma }_{{OHE}}{\overrightarrow{E}}^{2}$, where $\overrightarrow{s}$ and $\overrightarrow{L}$ are the spin and orbital angular momenta dynamically generated via the SHE and OHE, respectively (we replaced ${\vec{H}}_{{eff}}$ with $\overrightarrow{L}$). Such an interaction modulates the dynamical electrical conductivity, $\sigma$, which consists of two parts: $\sigma ={\sigma }_{0}+\gamma (\overrightarrow{s},\overrightarrow{L})$. Here, ${\sigma }_{0}$ is the samples’ electrical conductivity, and $\gamma (\overrightarrow{s},\overrightarrow{L})$ is the spin-dependent THz-induced contribution with $\gamma$ a scaling factor introduced to match the units. Combining Maxwell’s equations and the expression for the dynamical conductivity leads to a wave equation of the form (see Supplementary Materials for details)

1 $$\Delta \overrightarrow{E}+\frac{\varepsilon }{\mu {c}^{2}}\frac{{\partial }^{2}\overrightarrow{E}}{\partial {t}^{2}}=\frac{4\pi }{\mu {c}^{2}}{\sigma }_{0}\frac{\partial \overrightarrow{E}}{\partial t}+\frac{12\pi }{\mu {c}^{2}}\gamma {\sigma }_{{SHE}}{\sigma }_{{OHE}}{\overrightarrow{E}}^{2}\frac{\partial \overrightarrow{E}}{\partial t}$$

Equation(1) comprises two terms on its right side. The first term describes the screening effect of the fundamental radiation, while the second term relates to THG, with an amplitude proportional to the SHC. Note that the nonlinear interaction originating from $(\overrightarrow{s},\overrightarrow{s})$ or $(\overrightarrow{L},\overrightarrow{L})$ Hamiltonians could not describe the THz phase dependence on the SHC sign due to its positive (quadratic) nature.

While both spin and orbital angular momenta encounter nearly identical constraints in crystals, notable differences exist in their characteristic diffusion lengths and lifetimes. For instance, the spin diffusion length typically does not exceed 20 nm^15,45^, while the orbital diffusion length extends to 80 nm^21,43,44^. Similarly, spin (flip) relaxation time typically range in the hundreds of femtoseconds^45^, whereas orbital lifetimes are measured in picoseconds^21,43^. This justifies a potential *nonlocal dynamic* interaction between these degrees of freedom, which is incorporated in our $\gamma (\overrightarrow{s},\overrightarrow{L})$ term. The dynamic interaction of spin and orbital currents is not accounted for in our numerical simulations based on first principles. We further note that within our phenomenological framework, the magnitudes of spin and orbital currents exhibit linearity with respect to the amplitudes of the THz electric field, owing to the linear nature of both spin Hall and orbital Hall effects in TMs^21–25,28–30,40–44^. The nonlocal dynamic interplay between spin and angular momenta induces additional (spin dependent) electron scattering, consequently leading to THz field amplitude-dependent electrical conductivity, which results in odd harmonic generation in conducting materials. While both spin and orbital momenta are generated in the same metallic layer and by the same THz fields, the $\gamma (\overrightarrow{s},\overrightarrow{L})$ term is non-zero not only at the boundary but also in the bulk of the layer.

Our model is also able to explain the observed 60-fold increase in the THG intensity in the Pd film as the temperature decreases down to 5 K (as shown in Fig. 3C). The 4 nm thick metal layer residual resistivity ratio (RRR = (${\sigma }_{0}$(300 K)/($\,{\sigma }_{0}$(5 K)) was measured to be approximately 1.7, suggesting that the THG amplitude scales with ${\sigma }_{0}^{4}$. In the high resistivity regime, which is likely relevant for the sputter deposited 4 nm thin films^46,47^, both the intrinsic spin Hall and orbital Hall conductivities increase proportional to ${\sigma }_{0}^{2}$^30^. Hence, the scalar product of non-equilibrium spin and orbital momentum, arising from their dynamic coupling, leads to the modulation of THz THG amplitude with ${\sigma }_{0}^{4}$. Considering the consistent and relatively unchanged large OHC observed across all studied TM films, averaging around 4·10^3^ Ω^-1^cm^-1^ ^23,28–30^, the distribution of THG intensity across various samples correlates with their respective SHC characteristics.

In summary, our experiments establish a THz nonlinear response in the bulk of TM films, detectable through THz THG. These THG processes are observed across a broad range of metals, and we attribute them to spin-orbital coupling effects. Here, nonlocal dynamic interactions occur between THz-induced charge carriers and their spin and orbital degrees of freedom. The THz THG amplitude was found to correlate with the SHC, while its phase is determined by the sign of the spin Hall angle. These effects must be taken into account to understand the mechanisms of non-equilibrium and non-linear THz induced dynamics in transition metals on a microscopic level. Besides the fundamental aspects of nonlinear light-matter interactions in spin-orbit coupled systems, THz THG is of interest for practical applications, e.g. for the lithographic- and contact-free determination of ultrafast charge-spin interconversion, which forms the basis of emerging THz spintronics and THz orbitronics technologies.

## Methods

### Sample fabrication

The transition metal films were fabricated using dc magnetron sputter deposition at 3 × 10^−^^3 ^mbar Ar atmosphere in an ultrahigh-vacuum (4 × 10^−^^9 ^mbar) BESTEC system. We used 1-mm-thick double-side polished quartz (SiO_2_) substrates. A 10-nm-thick SiO_x_ layer, grown using rf sputter deposition, served as a cap layer to prevent the surface oxidation of the metallic films. All metallic layers were deposited at room temperature, with the use of a rotating sample holder to ensure the uniform growth of the metallic layers.

Ni_80_Fe_20_ and Fe films with a thickness of 4 nm were grown on single-crystalline MgO(001) substrates by molecular beam epitaxy (MBE) in a growth chamber with a base pressure below 3 × 10^−^^10 ^mbar at ambient temperature. Both films were capped by a 3 nm-thin Au layer. For the Ni_80_Fe_20_ we used electron beam evaporation, while Fe and Au were evaporated from an effusion cell with a flux of high stability. Before the deposition, the MgO substrates were preheated at 100 °C at a pressure of 4 × 10^−^^7 ^mbar for 2 h in the load lock chamber of the MBE system.

The thicknesses of all layers were controlled via the deposition time. Before the sample fabrication, the growth rate of each individual material was calibrated using X-ray reflectivity characterization of the corresponding film.

### Experimental setup

Time-domain spectroscopy was conducted utilizing both laser-based and accelerator-based terahertz sources. The laser-based THz source was based on a tilted pulse front generation (TPFG) scheme using a Ti:Sapphire laser system with 9 mJ pulse energy, 35 fs pulse duration at 800 nm central wavelength, operated at 1 kHz repetition rate. The THz pulses from TPFG scheme are initially broadband with frequency content spanning from 0.2 THz to 1.5 THz. Bandpass filters were employed for the conversion of broadband terahertz radiation into a narrowband spectrum. For temperature dependence of THz THG, we used the accelerator-based THz source TELBE located at Helmholtz-Zentrum Dresden-Rossendorf. The control of THz pump pulse polarization (polarization rotation and ellipticity) and the parameters of electro-optical detection scheme are similar as previously described in ref. ^48^. To control the polarization direction and/or amplitude of THz excitation a pair of rotatable wire grid polarizers have been used. To control the THz pulses ellipticity the waveplate based on x-cut crystalline quartz has been used. To compensate the polarization sensitivity of EOS, an additional pair of wire grid polarizers have been used^7^.

## Supplementary information

Supplementary Materials

## Data Availability

The data supporting this study and its findings are available within the article and Supplementary Information. All raw data can be provided by the authors upon reasonable request.
