# Supplementary material for: Spin-orbit interaction driven terahertz nonlinear dynamics in transition metals
Source: Npj Spintron. 2025 Jan 27;3(1):3. doi: 10.1038/s44306-024-00068-7 (PMC11772253; doi:10.1038/s44306-024-00068-7)
Supplement: Supplementary file 1 — Supplementary Materials [file 44306_2024_68_MOESM1_ESM.pdf]

Supplementary Materials for

Spin-orbit interaction driven terahertz nonlinear dynamics in transition metals

Ruslan Salikhov[1]*, Markus Lysne[2]*, Philipp Werner[2], Igor Ilyakov[1], Michael Schüler[2,3],Thales V. A. G. de Oliveira[1], Alexey Ponomaryov[1], Atiqa Arshad[1], Gulloo Lal Prajapati[1], Jan-Christoph Deinert[1], Pavlo Makushko[1], Denys Makarov[1], Thomas Cowan[1], Jürgen Fassbender[1,4], Jürgen Lindner[1], Aleksandra Lindner[1], Carmine Ortix[5], and Sergey Kovalev[1,6]*

*Corresponding author. Email: r.salikhov@hzdr.de, markus.lysne@unifr.ch, sergey.kovalev@tu-dortmund.de

The PDF file includes:

Supplementary Text
Figs. S1 to S11
Tables S1

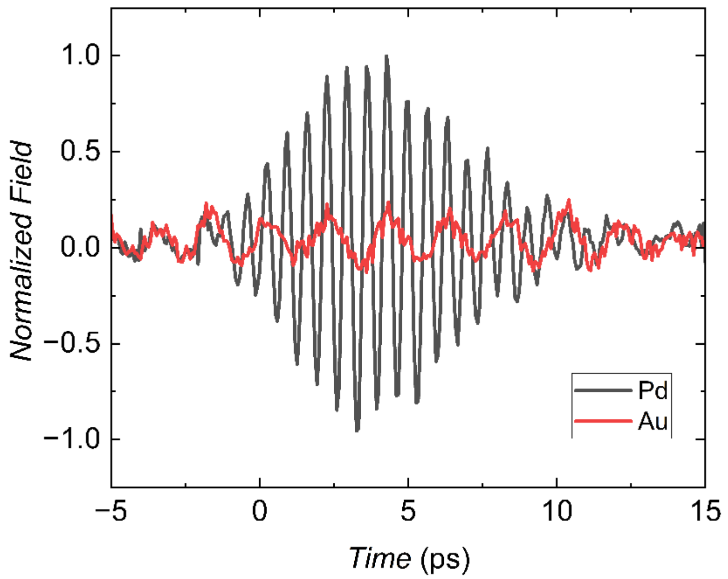

Fig. S1. Time domain scans of THz THG in 4 nm thick Pd and Au films. These data were recorded using two bandpass filters between the sample and the electro-optical sampling crystal. For further analysis, the contribution of the fundamental beam leak in the presented scans has been extracted using a digital high-pass filter. These data are shown in Fig. 1c.

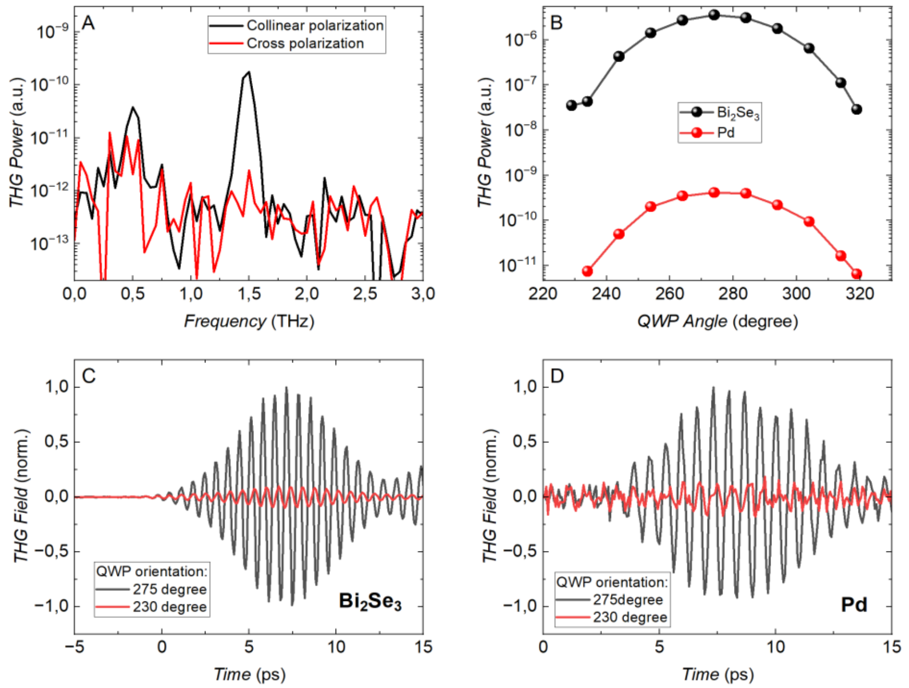

Fig. S2. THz THG polarization. (**A**) Comparison of the THG power for collinear and orthogonal polarization of the THG receiver with respect to the polarization of the fundamental beam. No THG signal is detected in the cross-polarization geometry. (**B**) Comparison of THG intensity in a 4-nm-thin Pd layer and Bi_2Se_3 film as a function of the pump beam ellipticity. The ellipticity is controlled by the orientation of the quarter-wave plate (QWP) for the incident THz beam. The THG efficiency in both films decreases with increasing pump beam ellipticity. At a QWP orientation of 275 degrees, the fundamental beam is linearly polarized. At 230 and 320 degrees of the QWP orientation, the fundamental beam becomes circularly polarized with opposite chirality (left or right). The level of THz THG in the Pd film measured with circularly polarized pump beams corresponds to the noise floor of the detection technique. The ellipticity dependence of THz THG in Bi_2Se_3 is similar to that previously reported in Ref. (*9*). (**C**) Time domain measurements of THz THG generated in Bi_2Se3 for QWP orientations at 230 and 275 degrees. (**D**) Time domain measurements of THz THG generated in the Pd sample for QWP orientations at 230 and 275 degrees.

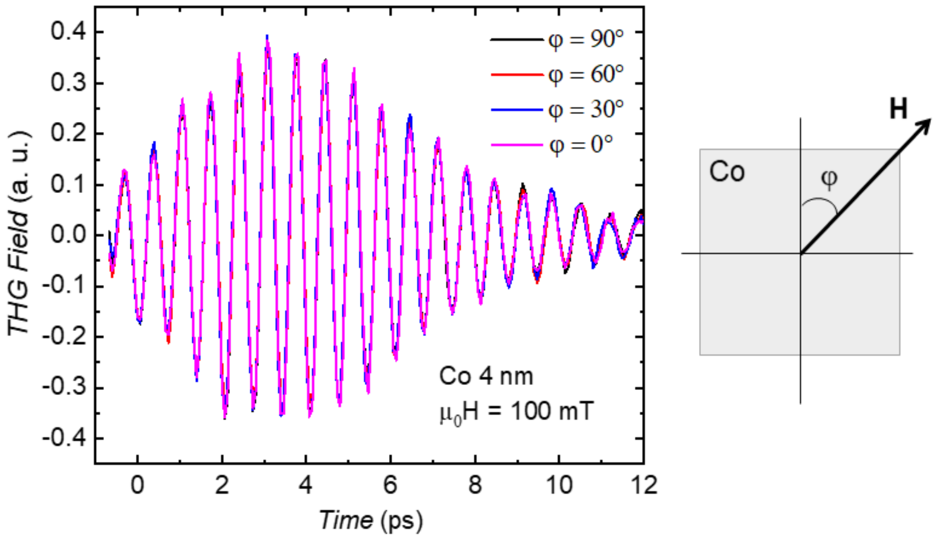

Fig. S3. THG in a ferromagnetic cobalt film. Comparison of the THG field amplitudes in a 4-nm-thin Co film for different angles of a 100 mT magnetic field applied parallel to the sample surface. φ = 0° corresponds to the polarization of the fundamental beam. The amplitude and phase of the THG are independent of the orientation of the Co magnetization with respect to the pump-pulse polarization. In the in-plane geometry, the Co film is magnetically saturated at a field of 100 mT.

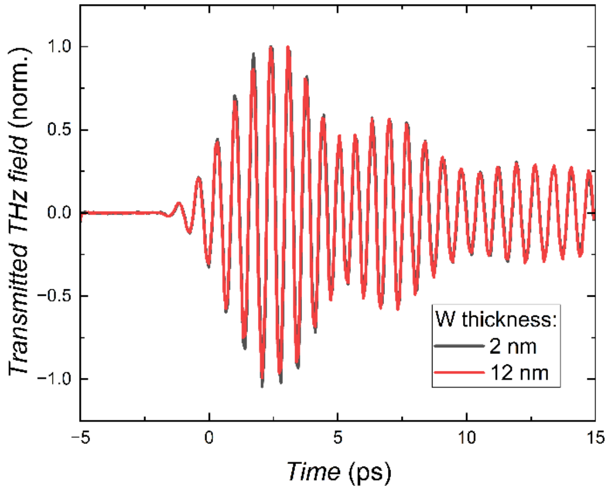

Fig. S4. Comparison of the time-domain THz signals obtained from 1.5 THz fundamental radiation transmitted through 2 nm and 12 nm thick W films. No dephasing in the transmitted radiation is observed with increasing W film thickness up to 12 nm within the experimental temporal resolution.

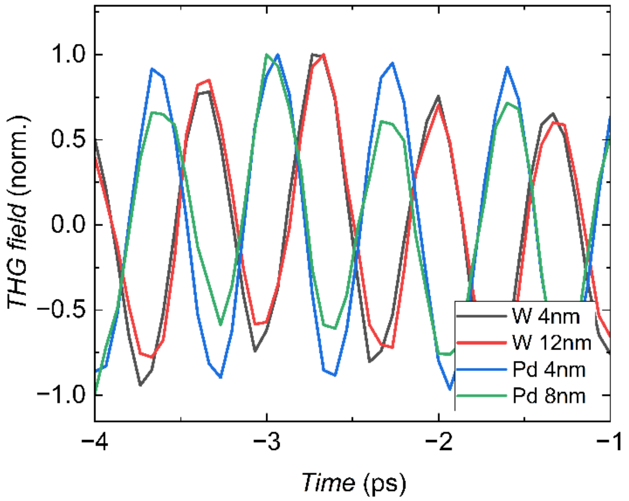

Fig. S5. THz third harmonic fields generated in W and Pd films of different thicknesses. All films were deposited on a 1-mm-thick quartz glass substrate and coated with a uniform thickness of SiO_x protection layer. It was observed that the THz THG phase remains consistent regardless of the metal thickness.

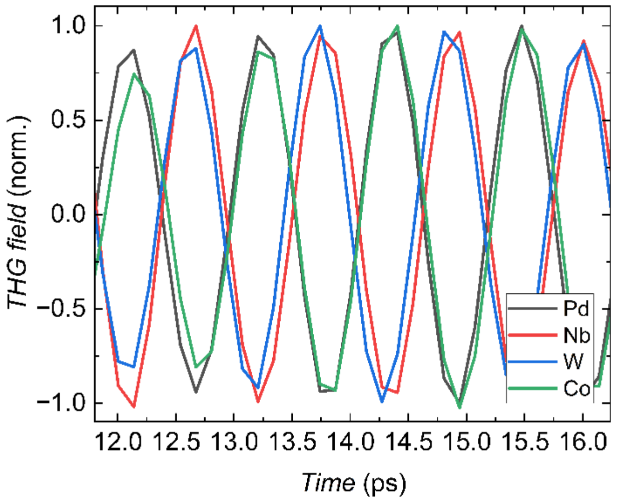

Fig. S6. THz third harmonic fields generated in 4 nm thick films of Pd, Nb, W and Co using 0.3 THz excitation. The THG frequency corresponds to 0.9 THz. It was observed that the THz THG phase depends on the *d*-shell filling in the same manner when either 0.3 THz or 0.5 THz excitation is used.

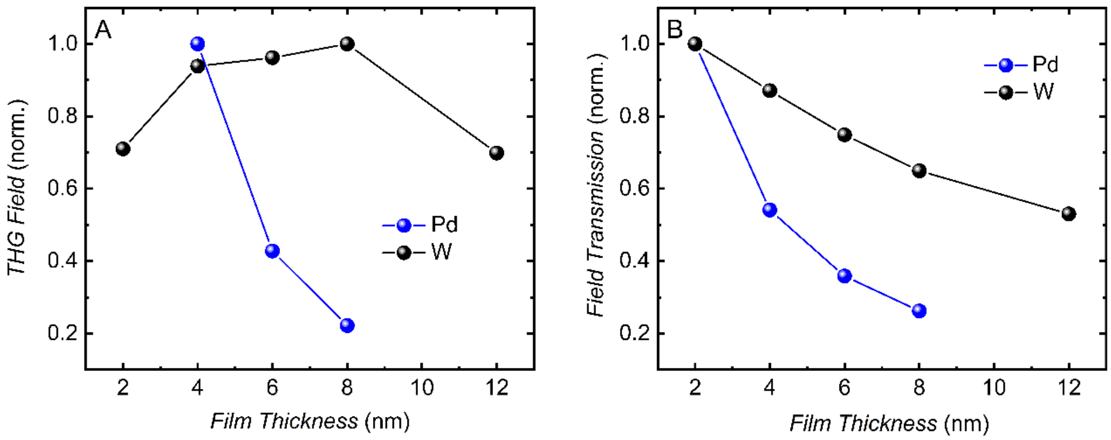

Fig. S7. Thickness dependence of THG. (**A**) Comparison of the THG field amplitude dependence on film thickness for Pd (blue circles) and W (black circles) films. Due to Pd's larger electrical conductivity and stronger screening effect, the THG signal in Pd samples decays faster with the film thickness than in W films. (**B**) Comparison of the transmission of metallic Pd (blue circles) and W (black circles) films for different film thicknesses. The transmitted 0.5 THz narrow-band radiation power is normalized to the maximum signal of the 2 nm Pd and W films.

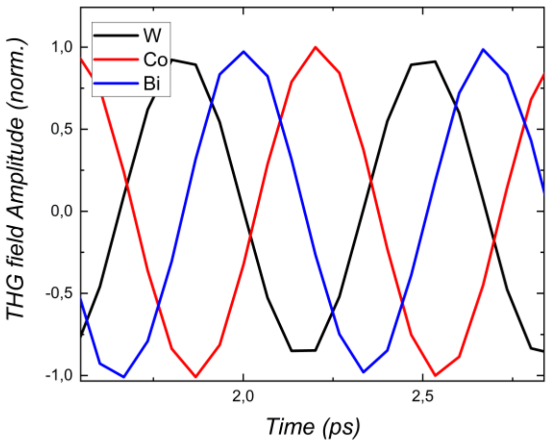

Fig. S8. THG phase in Bi and TM films. The Bi film was 25 nm thick (Co and W films – 4 nm) and was grown on the same substrate as the transition films. The THz THG in 25 nm Bi film is 50 times larger in amplitude than in Co film, but is absent in films with lower than 15 nm thickness.

Supplementary Text

Phenomenological model

As argued in the main text, we can qualitatively estimate the THz THG in TM by solving Maxwell's equations with a phenomenologically determined conductivity:

$$\begin{cases} rot\vec{E} = -\frac{1}{c}\frac{d\vec{B}}{dt} \\ rot\vec{B} = \frac{4\pi}{\mu c}\sigma\vec{E} + \frac{\varepsilon}{\mu c}\frac{d\vec{E}}{dt} \\ \sigma = \sigma_0 + \gamma\left(\vec{s}, \vec{L}\right) \end{cases} \quad (1)$$

Here, $\vec{E}$ and $\vec{B}$ are the electric and magnetic field of the THz pump pulses, c is the speed of light, σ is the total sample conductivity, σ_0 is the film electrical conductivity, and $\vec{s}$ and $\vec{L}$ are the spin and orbital angular momenta dynamically generated via the SHE and OHE, respectively. The dynamical spin distribution can be roughly approximated as $\vec{s} = \alpha_s\sigma_0\vec{E} \times \vec{r}$, where α_s is the spin Hall angle. The orbital momentum distribution is $\vec{L} = \alpha_o\sigma_0\vec{E} \times \vec{r}$, where α_o is the orbital Hall angle and $\vec{r}$ the radius vector. $\vec{L}$ and $\vec{s}$ have the same symmetry, being transverse to the electrical currents, and by averaging over the sample cross-section one gets $\left(\vec{s}, \vec{L}\right) \sim \alpha_s\alpha_o\sigma_0^2\vec{E}^2$. Solving Eq. (1) for the electric field we obtain the nonlinear wave equation

$$\Delta\vec{E} + \frac{\varepsilon}{\mu c^2}\frac{\partial^2\vec{E}}{\partial t^2} = \frac{4\pi}{\mu c^2}\sigma_0\frac{\partial\vec{E}}{\partial t} + \frac{12\pi}{\mu c^2}\gamma\alpha_s\alpha_o\sigma_0^2\vec{E}^2\frac{\partial\vec{E}}{\partial t} \ . \quad (2)$$

On the right side of Eq. (2) the first term describes the screening effect of the fundamental radiation. The second term describes the third harmonic generation process. The amplitude of the THz THG electric field is proportional to the product of $\alpha_s\alpha_o$. Since α_o does not change sign with d-band filling, while α_s changes the sign, the THz THG will have opposite polarity depending on the sign of SHC. The temperature dependence of THG demonstrates that the nonlinear process is more efficient at low temperatures, as seen in all investigated TM samples with a non-zero spin Hall conductivity.

One could argue that THG in TM could be attributed to intrinsic orbital current nonlinearity: the orbital currents generated by THz fields initially exhibit a third harmonic contribution, which is then converted back to charge currents through the inverse orbital Hall effect. However, this scenario does not explain the dependence of THG phase on the d-band filling, nor the correlation between THG amplitude and SHC. Additionally, the OHC should be significant for Nb metal (26), potentially resulting in a more intense THz THG compared to, for example, Pt, contrary to our experimental observation.

Numerical simulations

In order to gain insights into the microscopic mechanism behind the THG signal, we performed density functional theory (DFT) simulations. The calculations proceed by obtaining a relativistic band structure through DFT using the Quantum Espresso software package (*48*). DFT self-consistently determines the electronic charge density of a material by solving the effective single-particle problem

$$[-\frac{\hbar^2\nabla^2}{2m} + V_{eff}(r)]|\psi_{n\boldsymbol{k}}\rangle = \epsilon_n(\boldsymbol{k})|\psi_{n\boldsymbol{k}}\rangle, \tag{3}$$

where

$$V_{eff}(r) = V_{ion}(r) + V_H[n] + V_{XC}[n] \tag{4}$$

with $n(r) = \sum_{i\in N_{occc}} |\psi_i(r)|^2$. $V_{ion}(r)$ is the ionic (pseudo-)potential specifying the crystal structure of the material and $V_H[n]$ and $V_{XC}[n]$ are the Hartree and exchange potentials, respectively. In order to capture spin-orbit coupling effects, we use relativistic pseudopotentials in the DFT calculation.

Having obtained approximate Bloch wave functions of the material in question through DFT, we generate maximally localized Wannier functions using the Wannier90 library (*49*). This provides us with a set of maximally localized Wannier functions, $|\boldsymbol{R}a\rangle$, centered at site $\boldsymbol{R}$ in the crystal lattice. The corresponding matrix elements $\langle\boldsymbol{0}a|H|\boldsymbol{R}b\rangle$ enable us to formulate a tight-binding model for the material, and to incorporate the coupling to light in the dipole approximation:

$$H(t) = \sum_{\boldsymbol{k},ab} c^\dagger_{\boldsymbol{k}a}[T_{ab}(\boldsymbol{k} - q\boldsymbol{A}(t)) - q\boldsymbol{E}(t)\cdot\boldsymbol{D}_{ab}(\boldsymbol{k} - q\boldsymbol{A}(t))]c_{\boldsymbol{k}b}. \tag{5}$$

Here, $c_{\boldsymbol{k}a} = \frac{1}{\sqrt{N}}\sum_{\boldsymbol{R}} e^{i\boldsymbol{k}\boldsymbol{R}} c_{\boldsymbol{R}a}$, with $c_{\boldsymbol{R}a}$ the fermionic annihilation operator for an electron in Wannier orbital a at site $\boldsymbol{R}$, and

$$T_{ab}(\boldsymbol{k}) = \sum_{\boldsymbol{R}} e^{i\boldsymbol{k}\cdot\boldsymbol{R}}\langle\boldsymbol{0}a|H|\boldsymbol{R}b\rangle \tag{6}$$

the hopping term. $\boldsymbol{E}(t)$ is the electric field, which is related to the vector potential, $\boldsymbol{A}(t)$, by $\boldsymbol{E}(t) = -\partial_t\boldsymbol{A}(t)$. The dipole matrix elements are

$$\boldsymbol{D}_{ab}(\boldsymbol{k}) = \sum_{\boldsymbol{R}} e^{i\boldsymbol{k}\boldsymbol{R}}\langle\boldsymbol{0}a|(\boldsymbol{r} - \boldsymbol{R})|\boldsymbol{R}b\rangle. \tag{7}$$

For the materials considered, the dominant *s* and *d* character of the Wannier orbitals allows us to neglect the contribution from Eq. (7). If we neglect correlation effects beyond DFT, the time evolution of the density matrix ρ is governed by the Von-Neumann equation, which can be solved at each $\boldsymbol{k}$-point individually. Specifically,

$$\frac{d}{dt}\rho(\boldsymbol{k},t) = -i[T(\boldsymbol{k} - q\boldsymbol{A}(t)), \rho(\boldsymbol{k},t)] + D[\rho(\boldsymbol{k},t)], \tag{8}$$

where relaxation dynamics can be incorporated through the last term

$$D[\rho(\boldsymbol{k},t)] = -\frac{\rho(\boldsymbol{k},t) - \rho_{eq}(\boldsymbol{k},t)}{T_1} + (\frac{1}{T_1} - \frac{1}{T_2})\rho_{off}(\boldsymbol{k},t),$$

with $\rho_{eq}(\boldsymbol{k},t)$ the equilibrium density matrix and $\rho_{off}(\boldsymbol{k},t)$ the off-diagonal elements of $\rho(\boldsymbol{k},t)$ when expressed in the band basis of $T(\boldsymbol{k}-q\boldsymbol{A}(t))$ (*50*). Given $\rho(\boldsymbol{k},t)$, the charge current can be calculated as

$$\boldsymbol{J}(t)=\frac{1}{N}\sum_{\boldsymbol{k}}Tr[\rho(\boldsymbol{k},t)\nabla_{\boldsymbol{k}}T(\boldsymbol{k}-q\boldsymbol{A}(t))], \quad (9)$$

with N the number of $\boldsymbol{k}$-points in the Brillouin zone.

Whereas Eq. (8) with $D=0$ can be efficiently solved by unitary time evolution, the more general case must be solved by a Runge-Kutta method which is more computationally expensive because of the need for smaller time steps. Having tried various values of T_1, T_2, we find that the THG phase is not sensitive to these relaxation processes, and therefore opt for the unitary time evolution, which can be evaluated by means of a so-called commutator free expansion (*51*).

To determine the THG phase, we compute the Fourier transform

$$\boldsymbol{J}(\omega)=\int_{t_{min}}^{t_{max}}dte^{-i\omega t}\boldsymbol{J}(t),$$

where $[t_{min}, t_{max}]$ is the time interval of the electric field pulse. The THG signal can be studied in the time-domain by picking out the corresponding frequency component in the back-transformation, using a numerical approximation of the Dirac Delta function $\delta_a(\omega-3\Omega)$,

$$\boldsymbol{J}_{3\Omega}(t)=\frac{1}{\pi}\int_{\omega_{min}}^{\omega_{max}}d\omega e^{i\omega t}\boldsymbol{J}(\omega)\delta_a(\omega-3\Omega), \quad (10)$$

and taking its real part.

In order to illustrate the validity of our approximations - in particular using a basis of *s* and *d* orbitals - we present a band structure plot of Cobalt in Fig. S9(a), where we compare the DFT bands with those of the tight-binding model obtained from Wannier90. At least close to the Fermi energy, represented by a red horizontal dashed line, the tight-binding model provides an excellent fit. In panel (b), we show the spin texture from the Wannier90 data, which predicts the material to be magnetic. Including the same type of orbitals is found to give a very good fit also for the materials Nb, W, Pd and Ta.

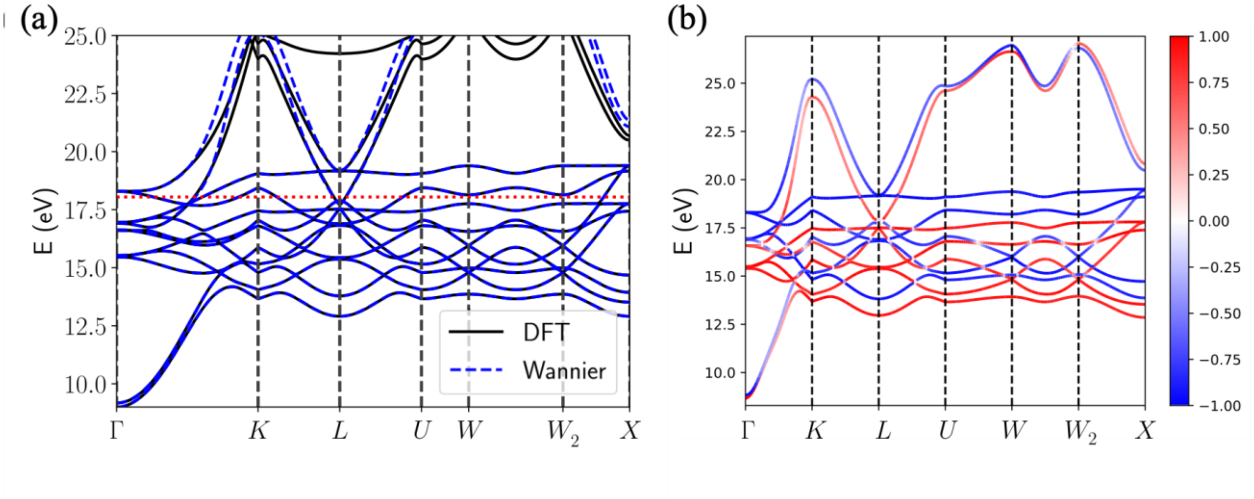

Fig. S9. Band structure. (**a**) Band structure of Co with an fcc crystal structure. The Fermi energy of 18.04 eV is marked by the red dotted line. The Wannierization considers *s* and *d* orbitals only. Co has an above half filled *d*-shell. In the Wannierization step, an inner window of [11,20] eV as well as an outer window of [0,40] eV has been used. (**b**) Spin texture of Co showing that Co is a magnetic material (the color-bar indicates the spin polarization).

Turning to the non-equilibrium dynamics, we begin by illustrating the effect of the relaxation time, T_1, for Pd in Fig. S10 for a rather small system size — a reciprocal space k-grid of 32^3 k-points. Notably, in the bottom panel, it is shown that the main effect of a shorter relaxation time is to enhance the non-linearities of the THG signal, while the THG phase is only slightly affected. This indicates that the question of the phase shift can be addressed using the dynamics obtained from the more efficient unitary time evolution.

We next present some results for the THG phase: Applying the above approximations, we find that at least for $F_0 = 8kV/cm$, the THG phase stays constant across all the materials considered. In a separate set of simulations, we found this to also hold for $F_0 = 2kV/cm$. The results for Pd and W are shown in Fig. S11.

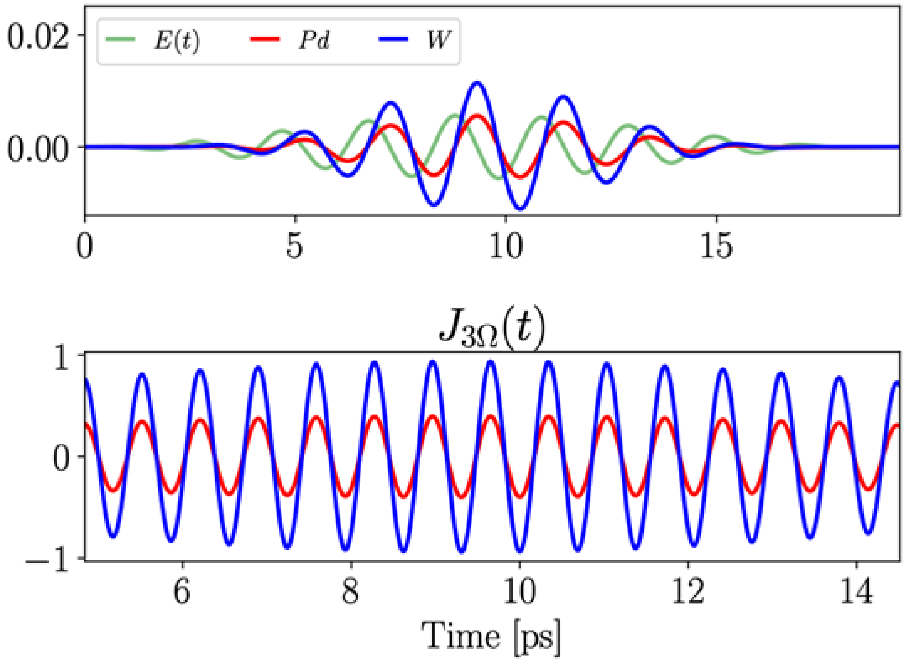

Fig. S10. Effect of relaxation. Top panel: Charge current for a 9 cycle laser pulse with $F_0 = 8kV/cm$ and period $T = 2ps$ using a sin^2 envelope. Red lines: $T_1 = 80fs, T_2 = 10fs$, blue lines: $T_1 = 200fs, T_2 = 10fs$. Bottom panel: THG signal computed from Eq. (10).

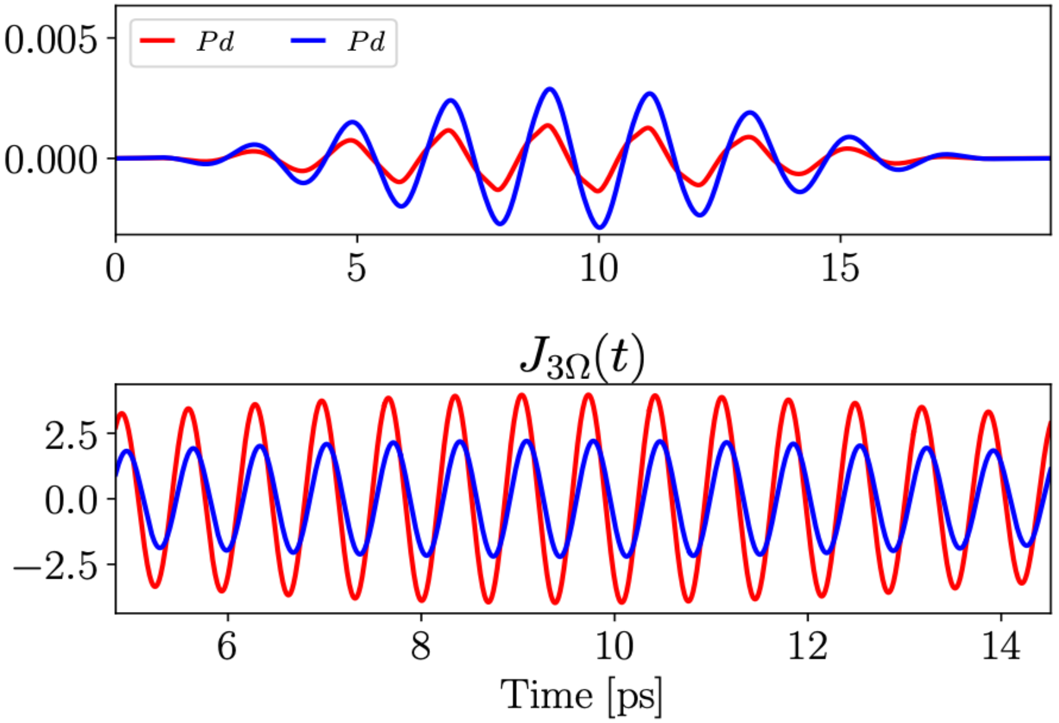

Fig. S11. THG phase of Pd and W. Top panel: Charge current for the 9-cycle laser pulse with $F_0 = 8kV/cm$ and period $T = 2\ ps$ using a sin^2 envelope. Bottom panel: The time evolution is computed by means of unitary time propagation. Bottom panel: third harmonic component for Pd (red) and W (blue).

That the numerical results do not show a 180 degree phase shift can be attributed to the approximations made in these simulations: Firstly, the coupling between the spin and orbital degree of freedom is inherent in the formalism, which should lead to a correction to the conductivity, as in Eq. (1) of the main text. What is however missing is the coupling to Maxwell's equations, which can give dynamical corrections to the electric field entering the ab-initio calculations. As a manifestation of this, we note that in order to avoid high-order non-linear responses, the simulations used $F_0 = 8kV/cm$, which is much smaller than the field strength used in the experiment. This large field strength reduction can be justified by the dynamical screening of the electric field, an effect which can either be modelled by a material dependent dielectric function or explicitly through Eq. (1) in the main text.

Regarding the effect of electron relaxation, which we have also studied, we found rather large enhancements of the non-linearities for smaller relaxation times. Since this effect was studied phenomenologically via the T1 and T2 parameters, the microscopic mechanisms behind the relaxation have not been accounted for, and may be highly material dependent. We leave more realistic studies of THG transmission as a future project. One possible avenue would be to simulate the dynamics through a Green's function formalism, using the Generalised Kadanoff Baym Ansatz, which incorporates the effects of electron-electron scattering (*52*).

Rashba-like surface states contribution

We first derive an effective Hamiltonian for surface states and to link its parameters to bulk physical quantities. To achieve this, we will use the theory of invariants that, following Ref. (*52*), we will then augment with atomic spin-orbit coupling. Let us start by considering surfaces of the materials in question with a $\boldsymbol{C_{3v}}$ point group symmetry, such as (111) surfaces. We choose surfaces with this symmetry since they are equipped with Berry curvature. We will also consider that the surface states are derived from $\boldsymbol{t_{2g}}$ electrons or, more generally, from a manifold that span an effective $\boldsymbol{L}$=1 subspace. Since we are considering a surface with $\boldsymbol{C_{3v}}$ point group symmetry, there are two generators of the point group: a vertical mirror symmetry, which, without loss of generality, we take as $\boldsymbol{M_x}$ sending $x \to -x$, and the threefold rotation symmetry with the rotation axis parallel to the mirror plane. The two generators can be therefore represented as

$$M_x = \begin{pmatrix} 1 & 0 & 0 \\ 0 & 1 & 0 \\ 0 & 0 & -1 \end{pmatrix};\ C_3 = \begin{pmatrix} 1 & 0 & 0 \\ 0 & \cos\frac{2\pi}{3} & \sin\frac{2\pi}{3} \\ 0 & -\sin\frac{2\pi}{3} & \cos\frac{2\pi}{3} \end{pmatrix},$$

Here we have chosen as basis states the well-known $|a_1\rangle = (|xy\rangle + |xz\rangle + |yz\rangle)/\sqrt{3}$ one-dimensional IRREP whereas the remaining two states form a two-dimensional IRREP given by $|e_\pm\rangle = (|xy\rangle + \omega^{\pm 1}|xz\rangle + \omega^{\pm 2}|yz\rangle)/\sqrt{3}$, with $\omega = e^{2\pi i/3}$. These states are thus formed by hybridization of the $|xy\rangle$, $|xz\rangle$ and $|yz\rangle$ orbitals in the presence of a planar triangular crystal field.

We can now uniquely determine the form of the effective Hamiltonian for the surface states using symmetry constraints. Specifically, we can use that any Hamiltonian for these states can be expanded in terms of the nine Gell-Mann matrices as

$$\mathcal{H}_{OR}(\boldsymbol{k}) = \sum_{i=0}^{8} b_i(\boldsymbol{k})\Lambda_i. \tag{1}$$

For convenience, we report at the end of this section the explicit form of the Gell-Mann matrices. The invariance of the Hamiltonian requires that the components of the Hamiltonian vector $\boldsymbol{b}(\boldsymbol{k})$ should have the same behavior as the corresponding Gell-Mann matrices Λ_i. This means that they should belong to the same representation of the crystal point group. From the representation of the Λ_i's [see Table 1] and those of the polynomials of $\boldsymbol{k}$ [see Table 1] and by further requiring time-reversal invariance, we find that the effective Hamiltonian reads

$$\mathcal{H}_{OR}(\boldsymbol{k}) = \Delta\left(\Lambda_3 + \frac{1}{\sqrt{3}}\Lambda_8\right) - \alpha_{OR}\left[k_x\Lambda_5 + k_y\Lambda_2\right] + \gamma(k_+^3 + k_-^3)\Lambda_7 + \frac{\hbar^2k^2}{2m}\Lambda_0\ . \tag{2}$$

Here the parameter Δ quantifies the crystal field splitting between the $|e_\pm\rangle$ doublet and the $|a_1\rangle$ singlet. The second term in the Hamiltonian above corresponds instead to a pseudo-spin one massless Dirac Hamiltonian and provides a linear coupling between the crystalline momentum $\boldsymbol{k}$ and the angular momentum $\boldsymbol{L}$: it is known as orbital Rashba coupling and has been shown to be relevant at transition metal oxide heterointerfaces (*53*). The third term in the equation above is a $\boldsymbol{C_{3v}}$-specific orbital warping term with $k_\pm = k_x \pm ik_y$. Finally, the last term is the kinetic energy term and has equal effective masses for the three orbitals. We point out that mass anisotropies are

in principle allowed and they can be accounted for via the doublet $(k_x^2 - k_y^2), k_x k_y$. For simplicity, we will neglect these terms since they will not change our conclusions.

C_{3v}	E	2 C_3	$2\sigma_v$	Polynomials of $\boldsymbol{k}$	Gell-Mann matrices
A_1	1	1	1	1, $k_x^2 - k_y^2$	$\Lambda_3 + \frac{\Lambda_8}{\sqrt{3}}, \Lambda_0$
A_2	1	1	-1	$k_x^3 + k_y^3$	Λ_7
E	2	-1	0	$\{k_x, k_y\}$	$\{\Lambda_1, \Lambda_4\}, \{\Lambda_2, \Lambda_5\}$ $\{\Lambda_6, \frac{\Lambda_3}{2} - \sqrt{3}\Lambda_8/2\}$

Table S1. Character table for the point group *C3v*.

Due to the presence of the orbital Rashba coupling, the Hamiltonian in Eq. (2) is characterized by a specific momentum-space orbital texture and therefore will display the so-called orbital Hall effect (see for instance Ref. (*40*)). However, it assumes *SU*(2) spin-rotation invariance and does not account for spin-orbit coupling. The latter can be included by treating spin and orbital degrees of freedom on an equal footing with the effective six-band surface Hamiltonian

$$\boldsymbol{\mathcal{H}_{tot}(k) = \mathcal{H}_{OR}(k) \otimes \sigma_0 + \lambda_{SO}(L_x \otimes \sigma_x + L_y \otimes \sigma_y + L_z \otimes \sigma_z)}. \quad (3)$$

Here $\boldsymbol{\sigma} = \{\boldsymbol{\sigma_x, \sigma_y, \sigma_z}\}$ is the usual Pauli matrix vector in spin space whereas $\boldsymbol{\lambda_{SO}}$ is the strength of the atomic spin-orbit coupling. Hund's third rule states that the sign of $\boldsymbol{\lambda_{SO}}$ discriminates between systems with less or more than half-filled *d* shells. Note also that in terms of Gell-Mann matrices we have that $\boldsymbol{L_x = \Lambda_2}$, $\boldsymbol{L_y = \Lambda_5}$ and $\boldsymbol{L_z = \Lambda_7}$. To proceed further, we consider the surface Hamiltonian at $\boldsymbol{k \equiv 0}$, which corresponds to the relevant time-reversal invariant point of the surface Brillouin zone, and diagonalize it. We thus move to an atomic spin-orbit coupled orbital basis that keeps explicitly track of the crystal field splitting. We then find three spin-orbit coupled Kramers' pairs with energies reading

$$\begin{aligned} \boldsymbol{E_1} &= -\frac{2\Delta}{3} + \lambda_{SO} \\ \boldsymbol{E_2} &= \frac{\Delta}{3} - \frac{\lambda_{SO}}{2} - \frac{1}{2}\sqrt{4\Delta^2 + 4\Delta\lambda_{SO} + 9\lambda_{SO}^2} \\ \boldsymbol{E_3} &= \frac{\Delta}{3} - \frac{\lambda_{SO}}{2} + \frac{1}{2}\sqrt{4\Delta^2 + 4\Delta\lambda_{SO} + 9\lambda_{SO}^2} \end{aligned} \quad (4)$$

A few remarks are in order here. First, in the spin-orbit-free limit, *i.e.* for $\boldsymbol{\lambda_{SO} \to 0}$, the Kramers' pair E_1 forms with the pair E_2 and E_3 respectively for $\Delta > \boldsymbol{0}$ and $\Delta < \boldsymbol{0}$ the $|\boldsymbol{e_\pm}\rangle$ quartet. Similarly when neglecting the crystal field, the Kramers' pair E_1 forms with the pair E_3 and E_2 respectively for $\boldsymbol{\lambda_{SO} > 0}$ and $\boldsymbol{\lambda_{SO} < 0}$ respectively, the total angular momentum *J*=3/2 quartet. Second, we point out that, as emphasized in Ref. (53), the existence of three distinct Kramers' pairs is consistent with a symmetry argument based on the double point group. This shows that in $\boldsymbol{C_{3v}}$ symmetric

crystals the spin-orbit coupled orbitals are either complex and stick together due to Kramers' theorem or are instead quaternionic and hence already equipped with time-reversal invariance.

We next write the eigenstates of the Kramers pairs as follows:

$$|\boldsymbol{v_{1+}}\rangle = [\mathbf{0},\mathbf{0},\mathbf{0},\mathbf{0},\boldsymbol{i},\mathbf{1}]/\sqrt{\mathbf{2}}$$
$$|\boldsymbol{v_{1-}}\rangle = [\mathbf{0},-\boldsymbol{i},\mathbf{1},\mathbf{0},\mathbf{0},\mathbf{0}]/\sqrt{\mathbf{2}}$$
$$|\boldsymbol{v_{2+}}\rangle = [\boldsymbol{f_2},\mathbf{0},\mathbf{0},\mathbf{0},-\boldsymbol{i},\mathbf{1}]/\sqrt{\mathbf{2}+\boldsymbol{f_2^2}}$$
$$|\boldsymbol{v_{2-}}\rangle = [\mathbf{0},\boldsymbol{i},\mathbf{1},-\boldsymbol{f_2},\mathbf{0},\mathbf{0}]/\sqrt{\mathbf{2}+\boldsymbol{f_2^2}}$$
$$|\boldsymbol{v_{3+}}\rangle = [\boldsymbol{f_3},\mathbf{0},\mathbf{0},\mathbf{0},-\boldsymbol{i},\mathbf{1}]/\sqrt{\mathbf{2}+\boldsymbol{f_3^2}}$$
$$|\boldsymbol{v_{3-}}\rangle = [\mathbf{0},\boldsymbol{i},\mathbf{1},-\boldsymbol{f_3},\mathbf{0},\mathbf{0}]/\sqrt{\mathbf{2}+\boldsymbol{f_3^2}}$$

where we introduced the factors $\boldsymbol{f_{2,3}} = \left(-\Delta - \boldsymbol{\lambda_{SO}}/\mathbf{2} \pm \sqrt{\mathbf{4}\Delta^2 + \mathbf{4}\boldsymbol{\lambda_{SO}} + \mathbf{9}\boldsymbol{\lambda_{SO}^2}/\mathbf{2}}\right)/\boldsymbol{\lambda_{SO}}$. We note that the two states of each pair are time-reversed partners as can be shown by applying the time-reversal operator $\boldsymbol{\mathcal{T}} = \boldsymbol{i\sigma_y}\otimes\boldsymbol{\Lambda_0}\boldsymbol{\mathcal{K}}$ with $\boldsymbol{\mathcal{K}}$ the complex conjugation. We now need to express the full Hamiltonian for spin and orbital degrees of freedom in this basis. Furthermore, as long as we are in the low-density regime for the surface states, we can neglect the mixing between the spin-orbit-split pairs of bands. In other words, the effective Hamiltonian can be written in a block form consisting of three $\mathbf{2}\times\mathbf{2}$ blocks. For the block related to the $|\boldsymbol{v_{1\pm}}\rangle$ time-reversed pairs we have $\boldsymbol{\mathcal{H}_{11}} = \boldsymbol{E_1} + \frac{\hbar^2\boldsymbol{k}^2}{\mathbf{2}\boldsymbol{m}} - \boldsymbol{\gamma}(\boldsymbol{k_+^3} + \boldsymbol{k_-^3})\boldsymbol{\sigma_z}$ with surface bands that thus have purely out-of-plane spin textures and a warping whose strength is not renormalized by atomic spin-orbit coupling. The surface Kramers' pairs related to $|\boldsymbol{v_{2+}}\rangle$ have instead the effective Hamiltonian

$$\boldsymbol{\mathcal{H}}_{22} = \boldsymbol{E}_2 + \frac{\hbar^2\boldsymbol{k}^2}{\mathbf{2}\boldsymbol{m}} + \boldsymbol{\gamma}\frac{\mathbf{2}\Delta + \boldsymbol{\lambda_{SO}} + \sqrt{\mathbf{4}\Delta^2 + \mathbf{4}\boldsymbol{\lambda_{SO}} + \mathbf{9}\boldsymbol{\lambda_{SO}^2}}}{\sqrt{\mathbf{4}\Delta^2 + \mathbf{4}\boldsymbol{\lambda_{SO}} + \mathbf{9}\boldsymbol{\lambda_{SO}^2}}}(\boldsymbol{k_+^3} + \boldsymbol{k_-^3})\boldsymbol{\sigma_z} - \boldsymbol{\alpha_R}\left(\boldsymbol{k_x}\boldsymbol{\sigma_y} + \boldsymbol{k_y}\boldsymbol{\sigma_x}\right) \quad (5)$$

where we have now obtained an intradoublet spin Rashba coupling given by

$$\boldsymbol{\alpha_R} = \frac{\mathbf{2}\boldsymbol{\alpha_{OR}}\boldsymbol{\lambda_{SO}}}{\sqrt{\mathbf{4}\Delta^2 + \mathbf{4}\boldsymbol{\lambda_{SO}} + \mathbf{9}\boldsymbol{\lambda_{SO}^2}}} \quad (6)$$

Finally, for the last pair of bands we have

$$\mathcal{H}_{22} = E_2 + \frac{\hbar^2 k^2}{2m} + \gamma \frac{-2\Delta - \lambda_{SO} + \sqrt{4\Delta^2 + 4\lambda_{SO} + 9\lambda_{SO}^2}}{\sqrt{4\Delta^2 + 4\lambda_{SO} + 9\lambda_{SO}^2}} (k_+^3 + k_-^3)\sigma_z + \alpha_R (k_x \sigma_y + k_y \sigma_x) \tag{7}$$

We note that the warping coefficient is not sign changing in terms of the atomic spin-orbit coupling coefficient λ_{SO}. On the contrary, the sign of the atomic spin-orbit coupling determines the sign of the surface Rashba coupling. Therefore, we can conclude by stating that surface states of materials with less or more than half-filled *d* shells can be discriminated by a relative π phase in the value of the Rashba coefficient.

Having established that the sign of λ_{SO} (and thus whether or not materials have less or more than half-filled shells) is reflected in the sign of the Rashba coefficient for the surface states, we next determine generic properties of non-linear current responses. In what follows we will concentrate exclusively on intraband processes and thus work in a semiclassical approximation. Let us start from second-order processes, which include both second harmonic generation and photogalvanic effects. As it is well known, the latter are only symmetry allowed in non-centrosymmetric systems. Furthermore, it is easy to see that at surfaces containing an evenfold rotation symmetry, the second-order responses are symmetry-forbidden. Hence, we can conclude that second-order responses are directly related to the Berry curvature. Each of the three Kramers related pairs of bands realize an effective two-level system for which the Berry curvature can be written as $\Omega^{\pm} = \pm d \cdot \left(\partial_{k_x} d \times \partial_{k_y} d\right)/2|d|^3$ where we introduced the d vector of the surface Hamiltonian $\mathcal{H}_{22} = d \cdot \sigma$. The sign change of the Rashba parameter corresponds to reverse two components of the d vector, which does not change the Berry curvature and thus second-order responses.

Let's now consider third-order current responses. It has been recently shown (*54*) that there are two different contributions to the third-order current response. The first one is directly related to the group velocity of the carriers - this is clearly insensitive to a sign change of the Rashba parameter since the surface dispersion is dependent on α_R^2. The second term is instead related to the so-called Berry connection polarizability tensor which can be related to the interband Berry connection $(\mathcal{A}_c)_{nm} = -i\langle u_n | \partial_{k_c} u_m \rangle$ via

$$\mathcal{G}_{ab} = 2Re \sum_{m \neq n} \frac{(\mathcal{A}_a)_{nm} (\mathcal{A}_b)_{mn}}{\varepsilon_n - \varepsilon_m} . \tag{8}$$

For a two-level system $m, n = \pm$. Furthermore, the wavefunctions of the two surface bands can be written in terms of the d vector components as $|u_{\pm}\rangle = \left[d_z \pm |d|, d_x - i d_y\right]^T /$

$\sqrt{2|\boldsymbol{d}|(|\boldsymbol{d}| \pm \boldsymbol{d_z})}$. Computation of the interband Berry connection in terms of the $\boldsymbol{d}$ vector components yields

$$(\mathcal{A}_c)_\pm = \frac{1}{2|d|\sqrt{|d|^2-d_z^2}}\left[d_x\partial_{k_c}d_y - d_y\partial_{k_c}d_x \pm i|d|\partial_{k_c}d_z \mp i d_z\partial_{k_c}|d|\right] . \quad (9)$$

We note that the interband Berry connection does not vanish in the absence of Berry curvature, *i.e.* for $\boldsymbol{d_z} \equiv \boldsymbol{0}$, thus implying that third-order nonlinear current responses will be finite even for conventional Rashba surface states. From the equation above, one also finds that the interband Berry connection is not sensitive to sign changes of the Rashba parameter $\boldsymbol{\alpha_R}$. In conclusion, we thus find that the sign of atomic spin-orbit coupling does not yield a $\boldsymbol{\pi}$ phase shift in the nonlinear current responses of surface states, neither at second order nor at third order.

Apart from the identity matrix $\boldsymbol{\Lambda_0}$, the eight Gell-Mann matrices can be defined as

$$\Lambda_1 = \begin{pmatrix} 0 & 1 & 0 \\ 1 & 0 & 0 \\ 0 & 0 & 0 \end{pmatrix}, \quad \Lambda_2 = \begin{pmatrix} 0 & -i & 0 \\ i & 0 & 0 \\ 0 & 0 & 0 \end{pmatrix},$$
$$\Lambda_3 = \begin{pmatrix} 1 & 0 & 0 \\ 0 & -1 & 0 \\ 0 & 0 & 0 \end{pmatrix}, \quad \Lambda_4 = \begin{pmatrix} 0 & 0 & 1 \\ 0 & 0 & 0 \\ 1 & 0 & 0 \end{pmatrix},$$
$$\Lambda_5 = \begin{pmatrix} 0 & 0 & -i \\ 0 & 0 & 0 \\ i & 0 & 0 \end{pmatrix}, \quad \Lambda_6 = \begin{pmatrix} 0 & 0 & 0 \\ 0 & 0 & 1 \\ 0 & 1 & 0 \end{pmatrix},$$
$$\Lambda_7 = \begin{pmatrix} 0 & 0 & 0 \\ 0 & 0 & -i \\ 0 & i & 0 \end{pmatrix}, \quad \Lambda_8 = \begin{pmatrix} \frac{1}{\sqrt{3}} & 0 & 0 \\ 0 & \frac{1}{\sqrt{3}} & 0 \\ 0 & 0 & -\frac{2}{\sqrt{3}} \end{pmatrix}.$$

Let us now check the properties of these eight Gell-Mann matrices under time-reversal symmetry. Since we are considering electrons that are effectively spinless due to the $SU(2)$ spin symmetry, the time-reversal operator can be represented as $\boldsymbol{\mathcal{K}}$. Hence, the three Gell-Mann matrices $\boldsymbol{\Lambda_2}, \boldsymbol{\Lambda_5}, \boldsymbol{\Lambda_7}$ are odd under time-reversal, *i.e.*, $\boldsymbol{\mathcal{T}^{-1}\Lambda_{2,5,7}\mathcal{T}} = -\boldsymbol{\Lambda_{2,5,7}}$, whereas the remaining matrices are even under time-reversal. Similarly, $\boldsymbol{\Lambda_{1,2,3,8}}$ are even under the vertical mirror symmetry whereas $\boldsymbol{\Lambda_{4,5,6,7}}$ are odd. Let us finally talk about the threefold rotational symmetry. Since the rotation symmetry operator $\boldsymbol{C_3} = \boldsymbol{exp}[\boldsymbol{2\pi i\Lambda_7/3}]$, the transformation properties of the

Gell-Mann matrices are determined by the commutation relations $[\boldsymbol{\Lambda_7}, \boldsymbol{\Lambda_i}]$. The commutation relations are as follows:

$$[\boldsymbol{\Lambda_7}, \boldsymbol{\Lambda_1}] = \boldsymbol{i\Lambda_4} \quad [\boldsymbol{\Lambda_7}, \boldsymbol{\Lambda_2}] = \boldsymbol{i\Lambda_4}$$
$$[\boldsymbol{\Lambda_7}, \boldsymbol{\Lambda_4}] = -\boldsymbol{i\Lambda_1} \quad [\boldsymbol{\Lambda_7}, \boldsymbol{\Lambda_5}] = -\boldsymbol{i\Lambda_2}$$
$$[\boldsymbol{\Lambda_7}, \boldsymbol{\Lambda_6}] = \boldsymbol{2i}\left(\frac{\boldsymbol{\Lambda_3}}{\boldsymbol{2}} - \frac{\sqrt{\boldsymbol{3}}}{\boldsymbol{2}}\boldsymbol{\Lambda_8}\right) \quad \left[\boldsymbol{\Lambda_7}, \frac{\boldsymbol{\Lambda_3}}{\boldsymbol{2}} - \frac{\sqrt{\boldsymbol{3}}}{\boldsymbol{2}}\boldsymbol{\Lambda_8}\right] = -\boldsymbol{i\Lambda_6}$$
$$\left[\boldsymbol{\Lambda_7}, \frac{\boldsymbol{\Lambda_3}}{\boldsymbol{2}} + \frac{\boldsymbol{\Lambda_8}}{\sqrt{\boldsymbol{3}}}\right] = \boldsymbol{0}$$

The results above indicate that the three pairs of operators$\{\boldsymbol{\Lambda_1}, \boldsymbol{\Lambda_4}\}$, $\{\boldsymbol{\Lambda_2}, \boldsymbol{\Lambda_5}\}$, and $\left\{\boldsymbol{\Lambda_6}, \frac{\boldsymbol{\Lambda_3}}{\boldsymbol{2}} - \frac{\sqrt{\boldsymbol{3}}}{\boldsymbol{2}}\boldsymbol{\Lambda_8}\right\}$ behave as a vector under the threefold rotation symmetry and therefore form two-dimensional IRREPS.

[48] P. Giannozzi, et al., QUANTUM ESPRESSO: a modular and open-source software project for quantum simulations of materials. *J. of Physics: Condens. Matter* **21**, 395502 (2009). https://iopscience.iop.org/article/10.1088/0953-8984/21/39/395502/meta

[49] G. Pizzi, et al., Wannier90 as a community code: new features and applications. *J. Phys.: Condens. Matter* **32**, 165902 (2020). https://iopscience.iop.org/article/10.1088/1361-648X/ab51ff/meta

[50] M. Schüler, Y. Murakami, Doping and gap size dependence of high-harmonic generation in graphene: Importance of consistent formulation of light-matter coupling. *Phys. Rev. B* **106**, 035204 (2022). https://doi.org/10.1103/PhysRevB.106.035204

[51] A. Alvermann, H. Fehske, High-order commutator-free exponential time-propagation of driven quantum systems. *Journal of computational physics* **230**, 5930-5956 (2011), Pages. https://doi.org/10.1016/j.jcp.2011.04.006

[52] P. Lipavský et al., Generalized Kadanoff-Baym ansatz for deriving quantum transport equations. *Phys. Rev. B* **34**, 6933 (1986). https://journals.aps.org/prb/abstract/10.1103/PhysRevB.34.6933

[53] E. Lesne et al., Designing spin and orbital sources of Berry curvature at oxide interfaces. Nature Materials **22**, 576 (2023). https://doi.org/10.1038/s41563-023-01498-0

[54] H. Liu et al., Berry connection polarizability tensor and third-order Hall effect. Phys. Rev. B **105**, 045118 (2023). https://doi.org/10.1103/PhysRevB.105.045118
